# A multimarker QPCR-based platform for the detection of circulating tumour cells in patients with early-stage breast cancer

**DOI:** 10.1038/bjc.2011.164

**Published:** 2011-05-17

**Authors:** T J Molloy, L A Devriese, H H Helgason, A J Bosma, M Hauptmann, E E Voest, J H M Schellens, L J van't Veer

**Affiliations:** 1 Division of Experimental Therapy, The Netherlands Cancer Institute, Plesmanlaan 121, 1066 CX Amsterdam, The Netherlands; 2 Department of Clinical Pharmacology, The Netherlands Cancer Institute, Plesmanlaan 121, 1066 CX Amsterdam, The Netherlands; 3 Department of Bioinformatics and Statistics, The Netherlands Cancer Institute, Plesmanlaan 121, 1066 CX, Amsterdam, The Netherlands; 4 Department of Medical Oncology, University Medical Center Utrecht, P.O. Box 85500, 3508 GA, Utrecht, The Netherlands; 5 Science Faculty, Department of Pharmaceutical Sciences, Utrecht University, P.O. Box 80082, 3508 TB Utrecht, The Netherlands

**Keywords:** early-stage breast cancer, circulating tumour cell, CTC, enrichment, quantitative PCR, prognosis

## Abstract

**Background:**

The detection of circulating tumour cells (CTCs) has been linked with poor prognosis in advanced breast cancer. Relatively few studies have been undertaken to study the clinical relevance of CTCs in early-stage breast cancer.

**Methods:**

In a prospective study, we evaluated CTCs in the peripheral blood of 82 early-stage breast cancer patients. Control groups consisted of 16 advanced breast cancer patients and 45 healthy volunteers. The CTC detection was performed using ErbB2/EpCAM immunomagnetic tumour cell enrichment followed by multimarker quantitative PCR (QPCR). The CTC status and common clinicopathological factors were correlated to relapse-free, breast cancer-related and overall survival.

**Results:**

Circulating tumour cells were detected in 16 of 82 (20%) patients with early-stage breast cancer and in 13 out of 16 (81%) with advanced breast cancer. The specificity was 100%. The median follow-up time was 51 months (range: 17–60). The CTC positivity in early-stage breast cancer patients resulted in significantly poorer relapse-free survival (log rank test: *P*=0.003) and was an independent predictor of relapse-free survival (multivariate hazard ratio=5.13, *P*=0.006, 95% CI: 1.62–16.31).

**Conclusion:**

The detection of CTCs in peripheral blood of early-stage breast cancer patients provided prognostic information for relapse-free survival.

Breast cancer mortality rates have declined over the last decade because of better screening and improved diagnostic techniques and treatments; however, it still remains the main cause of cancer-related deaths in women worldwide (Levi *et al*, 2005). Approximately one-third of all women with primary breast cancer will develop metastatic disease (Rosen *et al*, 1989; Fisher *et al*, 2002), whereby the risk of relapse is strongly related to lymph node involvement, tumour size, grade at diagnosis (Fitzgibbons *et al*, 2000), lymphatic and/or vascular invasion, hormone receptor status and presence of HER2 overexpression (Pinder *et al*, 1994). More recently, the risk of metastasis has also been shown to correlate well with prognostic gene expression profiles (van de Vijver *et al*, 2002; Paik *et al*, 2004; Wang *et al*, 2005).

The detection of circulating tumour cells (CTCs) has been correlated to poor progression-free survival in patients with metastatic breast cancer (Cristofanilli *et al*, 2004, 2005). For CTCs, starting with peripheral blood as sampling material is advantageous for the patient, as it is much less invasive to obtain than a tumour biopsy. Moreover, the accessibility enables sequential sampling during therapy. In patients with early-stage breast cancer, the detection of CTCs or disseminated tumour cells (DTCs) in the blood and/or bone marrow has also been found to be an independent negative prognostic factor for disease recurrence and overall survival (Mansi *et al*, 1999; Pantel *et al*, 1999; Braun *et al*, 2000). However, relatively few studies have been undertaken to investigate the prognostic significance of CTCs in non-metastatic breast cancer patients (Daskalaki *et al*, 2009; Muller and Pantel, 2009; Xenidis *et al*, 2009), perhaps because of the high assay sensitivity and specificity required for such studies as a result of the relative rarity of CTCs.

Previously, we designed a quantitative PCR (QPCR)-based assay that utilises a panel of four tumour marker genes for the detection of occult tumour cells in the peripheral blood of metastatic breast cancer patients. The tumour marker genes had been selected after a systematic search for genes that are highly expressed in breast cancer, but not in the cellular constituents of peripheral blood (Bosma *et al*, 2002). Our test showed a sensitivity of 31% and a specificity of 100% in metastatic breast cancer patients and predicted for a worse progression-free and overall survival (Weigelt *et al*, 2003). Next, we optimised the assay's sensitivity by introducing a dual-antigen immunomagnetic tumour cell enrichment procedure before marker gene quantitation and by refining the panel of marker genes as follows: *cytokeratin 19* (*CK19*), *human secretory protein p1.B* (*p1B*), *human epithelial glycoprotein* (*EpCAM*; here: *EGP*) and *mammaglobin* (*MmGl*) (Molloy *et al*, 2008). In spiking experiments, we showed that our assay has the sensitivity of detecting as few as 10 tumour cells from a background of 10^6^ peripheral blood mononuclear cells (PBMCs) (Molloy *et al*, 2008).

In this study, we used our improved platform for CTC detection in a prospective cohort of patients with early-stage breast cancer, in an effort to detect the presence of CTCs at diagnosis. These data were then correlated to disease outcome. In addition, we used our platform in two control groups, being advanced breast cancer patients and healthy volunteers.

## Materials and methods

The methods and data described herein adhere to the REMARK criteria for the reporting of tumour marker prognostic studies (McShane *et al*, 2006).

### Patient selection and peripheral blood sampling

Written informed consent was obtained from all participants and the study was approved by the medical ethical committee of the Netherlands Cancer Institute. Women presenting at the outpatient clinic of The Netherlands Cancer Institute with clinically stage I–III breast cancer were invited to participate between May 2005 and May 2006. Patients with a history of previous malignancy and patients with disseminated breast cancer or active infection were excluded. Type of surgery, locoregional radiotherapy and adjuvant systemic therapy was left to the treating physician, following nationwide standardised protocols.

From all patients, 8.0 ml whole blood samples were collected during routine preoperative blood sampling in tubes containing a Ficoll-Hypaque density fluid separated by a polyester gel barrier from a sodium citrate anticoagulant (Vacutainer CPT, Beckton Dickinson, Breda, The Netherlands). Mononuclear cells, including any tumour cells present, were isolated from blood samples within 24 h of collection.

### Selection of advanced breast cancer patients and healthy volunteer control subjects

Patients with advanced breast cancer (M1 disease, according to the Union Internationale Contre le Cancer criteria) were included as ‘positive controls’ as the majority were expected to have CTCs. They were invited to participate if they were between treatments or soon to start subsequent palliative treatment modality. Also, a third group of healthy, female anonymous control subjects, who were randomly selected from hospital staff, were asked to participate. Blood sample collection and preparation were performed as described above.

### Additional analyses: effect of frozen storage and the value of repeated sampling after therapy

In order to assess the effect of frozen storage, samples of a subgroup of patients and healthy volunteers were collected in duplicate. Mononuclear fractions were isolated and one sample was analysed directly and the other was supplemented with ‘freezing medium’ (RPMI-1640 medium with L-glutamine (Gibco, Breda, The Netherlands) containing 10% dimethylsulphoxide, (DMSO; Merck, Darmstadt, Germany) and 20% fetal calf serum (Gibco) and stored in liquid nitrogen. After 3 months, the frozen pellets were thawed and enriched as described above.

In order to gain insight into the additional value of our assay after therapy, peripheral blood samples were collected between October 2008 and February 2009, after written informed consent, from a subset of patients who continued to visit the outpatient clinic and had remained disease free.

### Tumour cell enrichment

Tumour cells were separated from PBMCs using anti-EpCAM (CD326) (clone HEA-125) and anti-ErbB2 (HER2) Micro Beads (MACS, Miltenyi Biotec, Utrecht, The Netherlands) according to the manufacturer's instructions. In brief, beads were incubated with the PBMCs for 30 min at 4 °C, after which labelled cells were collected on a magnetic separation column. After removal of the column from the magnetic field, the retained EpCAM^+^ and/or ErbB2^+^ cells were eluted, and stored at −70 °C in lysis buffer (5 M Guanidine thiocyanate (Merck), pH 6.8, 0.05 M Tris (Roche, Mannheim, Germany), 0.02 M EDTA and 1.3% Triton X-100 (Sigma, Steinheim, Germany)) until mRNA isolation and cDNA synthesis.

### mRNA isolation and cDNA synthesis

mRNA was precipitated from the cell lysate and dissolved in lysis buffer from the *μ*MACS One-step cDNA kit (Miltenyi Biotec). Oligo(dT) Micro Beads were added and the mixture placed onto the *μ*MACS column in the thermo MACS Separator. Next, cDNA synthesis was carried out as per the manufacturer's instructions, with an additional elution with 20 *μ*l of elution buffer, resulting in a total volume of 70 *μ*l.

### Quantitative real-time PCR

Quantitative real-time PCR primers (Sigma Genosys, Cambridge, UK) and 5′-fluorescently FAM-labelled TaqMan probes (Applied Biosystems, Nieuwerkerk a/d IJssel, The Netherlands) were designed using the Perkin Elmer Primer Express software (PE, Foster City, CA, USA) based on the published sequences of *CK19*, *p1B*, *EGP* and *MmGl* as previously described (Molloy *et al*, 2008) (Table 1). All primers were designed to be intron-spanning to preclude amplification of genomic DNA. Commercially available primers and probes for the ‘housekeeping’ genes *β-actin* and *glyceraldehyde-3-phosphate dehydrogenase* (*GAPDH*) (Applied Biosystems) were also used.

Serially diluted cDNA synthesised from the amplified RNA of 82 snap frozen breast cancer tissues was used to generate standard curves for control and marker gene expression. For all cDNA dilutions, fluorescence was detected from 0 to 50 PCR cycles for the control and marker genes in singleplex reactions, which allowed the deduction of the C_T_ value for each product. The C_T_ value (threshold cycle) is the PCR cycle at which a significant increase in fluorescence is detected because of the exponential accumulation of PCR products and is represented in arbitrary units (TaqMan Universal PCR Master Mix Protocol, Applied Biosystems) (Bieche *et al*, 1999). The expression of each tumour marker gene was calculated relative to *β*-*actin* in each sample, and the second ‘housekeeping’ gene, *GAPDH*, was used only to confirm reaction efficiency. Each experiment was performed in triplicate. Quality control measures for the PCR reactions included the addition of a genomic DNA control and a non-template control.

### Clinical follow-up

Of every patient, up to 1 February 2010, regular clinical follow-up was recorded in the patient file and the Institute's Medical Registry. This included evaluation of relapse of disease, breast cancer-related death and death by other causes. Relapse of disease was defined as the development of either local or distant breast cancer metastases. In case of relapse, the date of diagnosis was recorded. In the absence of relapse, the date of the last visit to the outpatient clinic within a year before February 2010 was recorded as last follow-up date. When needed, for example, when the last follow-up was longer than a year before or further treatment took place in another hospital, information was verified with the general physician and the last visit there was recorded as last follow-up. One patient without relapse refused further participation after 44 months of follow-up, being longer than a year before February 2010. This date was recorded as last follow-up and censored for further analysis.

### Statistics

Analyses were carried out using SPSS version 17.0 (SPSS Inc., Chicago, IL, USA). All statistical tests were performed at the 5% level of significant difference. Differences of rates between groups were compared with either the two-sided Fisher's exact test or Pearson's *χ*^2^-test or, for ordinal variables, *χ*^2^-test for trend. Differences between groups with continuous variables were tested by the nonparametric Mann–Whitney *U-*test. The quadratic discriminant analysis (QDA) score function was calculated from the expression data of four marker genes (*CK19*, *p1B*, *EGP* and *MmGl*) as previously described (Hand, 1992; Bosma *et al*, 2002). The QDA is a statistical approach to find the combination of quadratic and linear functions of variables (in this case marker genes), which leads to the optimal separation between groups (in this case, advanced breast cancer patients and healthy volunteers). It is a generalisation of the more familiar Fisher's linear discrimination analysis (LDA), which allows only linear functions (Weigelt *et al*, 2004). The highest value of the healthy control group was set as threshold value for positivity (QDA value > threshold: ‘QDA-positive’) and negativity (for QDA value ⩽ threshold: ‘QDA-negative’) of other samples. This threshold was fixed for any future study. QDA positivity indicated the presence of tumour cells in a sample and, conversely, QDA negativity indicated their absence. Patients with a QDA-positive or QDA-negative test from their blood sample were defined as having a positive or negative CTC status, respectively. The QPCR measurement and QDA data analysis in this way offers a simple and objective estimate of tumour cell presence in a given sample. Survival was illustrated by Kaplan–Meier plots and compared between groups by the log rank test. Clinicopathological factors known to be associated with prognosis (age ⩽45 years *vs* >45 years), tumour size (T2 and T3 *vs* T1), lymph node involvement (yes *vs* no), both following TNM 6 classification according to Union Internationale Contre le Cancer criteria (Sobin and Wittekind, 2002), histological grade (3 *vs* 1 or 2), oestrogen receptor (ER) and/or progesterone receptor (PR) hormone receptor (both negative *vs* either or both positive) and HER2 (positive=3+ in immunohistochemistry or in fluorescent *in situ* hybridisation *vs* negative) were tested in univariate analysis to calculate hazard ratios (HRs), their 95% confidence intervals (CIs) and *P*-value. Variables that were found to be significant or with the HR >2.0 or <0.5 in the univariate analysis were included in a multivariate Cox regression model to identify those with independent prognostic information and furthermore to calculate HRs and their 95% CIs.

## Results

### Study inclusion and patient characteristics

A total of 82 women with early-stage breast cancer were included and a peripheral blood sample was obtained before surgery and before initiating adjuvant therapy. The clinical characteristics are described in Table 2. The median age was 56 years (range: 34–86 years), 70 (85%) were >45 years, 12 (15%) had stage III disease at diagnosis, 20 (24%) had tumours >2 cm, 22 (27%) had infiltrated axillary lymph nodes, 18 (22%) had tumours with histological grade 3, 9 (11%) had tumours that were ER and PR negative and 28 (22%) had tumours that were HER2 positive. In total, 68 (83%) of the patients received any type of adjuvant therapy and 14 (17%) received trastuzumab therapy. The median time from diagnosis to blood collection was 14 days (range: 0.0–61 days) and the median follow-up time from sampling to ‘relapse of disease’ or ‘last follow-up’ was 51 months (range: 17–60). The median follow-up time for patients who did not experience an event was 51 months (range: 40–60).

### Circulating tumour cell detection in patient groups

**Figure 1:**
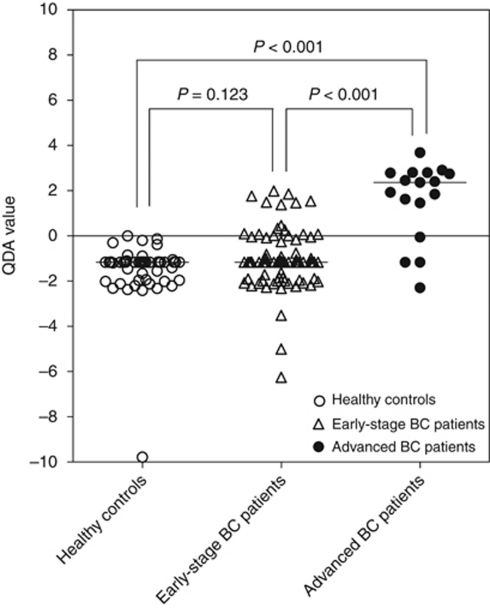
Quadratic discriminant analysis (QDA) values incorporating the expression of the four marker genes *CK19*, *p1B*, *EGP* and *MmGl* measured in the peripheral blood of healthy controls (*n*=45; open circles), early-stage breast cancer (BC) patients (*n*=82; triangles) and advanced BC patients (*n*=16; closed circles). The median expression levels for the QDA are indicated by a horizontal line (healthy controls=−1.16, early-stage BC patients=−1.16, advanced breast cancer patients=2.39).

In this prospective study, 16 (20%) of the 82 primary breast cancer patients who were included had a positive QDA score (Figure 1). The majority of patients with a QDA-positive score (12 out of 16; 75%) had a blood sample that was positive for the *EGP* marker gene and at least one other marker gene (5 out of 16, 31% was *EGP* and *p1B* positive; 4 out of 16, 25% was *EGP* and *CK19* positive; and 3 out of 16, 19% was *EGP*, *p1B* and *CK19* positive). The four other QDA-positive patients (4 out of 16, 25%) had a blood sample that was *EGP* negative but positive for both *p1B* and *MmGl*. The sensitivity and specificity of our test was 20% (95% CI: 12–30) and 100% (95% CI: 92–100), respectively. The distribution of patient and primary tumour tissue characteristics were not significantly different between the CTC-positive and CTC-negative patients (Table 2). The median age at diagnosis was 55 (range: 36–84) and 57 (range: 34–86) for the CTC-positive and CTC-negative patients, respectively. In addition to this, a female healthy volunteer control group (*n*=45, none positive) and advanced breast cancer patients group (*n*=16; 13 (81%) positive) were tested (Figure 1). The QDA values among advanced breast cancer patients were higher compared with early breast cancer patients as well as with healthy controls (Mann–Whitney *U-*test: both *P*<0.001), although there was no significant difference between early-stage patients and healthy controls (*P*=0.123). Median QDA values (range) were −1.16 (−9.78 to 0.00), −1.16 (−6.25 to 1.99) and 2.39 (−1.16 to 3.69) for the healthy controls group, early-stage breast cancer patients and advanced breast cancer patients, respectively. For further analysis, patients with a zero or negative QDA were considered ‘CTC-negative’ and patients with a positive QDA were considered ‘CTC-positive’.

### CTC status at the time of diagnosis and clinical outcome

During follow-up period of the early breast cancer patients, 12 patients (14%) experienced clinical relapse: 6 of whom were CTC positive (38% of all CTC-positive patients), and 6 of whom were CTC negative (9% of all CTC-negative patients; Fisher's exact test: *P*=0.010; Table 3). One CTC-positive patient had a local relapse, followed by distant metastasis 1 month later. The other 11 patients had distant metastases in pleura, liver, brain, bone or in multiple sites.

**Figure 2:**
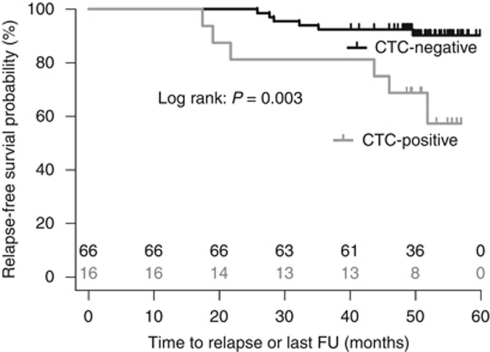
Kaplan–Meier survival curve for relapse-free survival of early-stage breast cancer patients (*n*=82) who were CTC negative (*n*=66) or positive (*n*=16) at diagnosis. CTC-positive patients had a significantly poorer relapse-free survival than CTC-negative patients (univariate hazard ratio=4.72; 95% CI: 1.52–14.66; log rank test *P*=0.003). The number of patients at risk at each time point (months) are indicated for the CTC-negative (black) and CTC-positive (grey) groups. Abbreviations: CTC-positive or CTC-negative=positive or negative circulating tumour cell status according to quadratic discriminant analysis (QDA) score; FU=follow-up.

Despite the relatively short follow-up period of this prospective study, CTC status at the time of diagnosis as determined by our assay was a significant predictor of relapse-free survival, with a hazard ratio of 4.72 (95% CI: 1.52–14.66, *P*=0.003; Figure 2 and Table 4). Importantly, multivariate analysis demonstrated that CTC status at the time of diagnosis was a significant and independent predictor of relapse-free survival (multivariate Cox regression, multivariate hazard ratio=5.13, *P*=0.006, 95% CI: 1.62–16.31; Table 5). The 4-year relapse-free survival rates were 92 and 69% for CTC-negative and CTC-positive patients, respectively (Figure 2).

During the follow-up period, death occurred in total in eight patients, of which five were breast cancer related and three were by other causes (Table 3). One death occurred in the CTC-positive group and this was a breast cancer-related death (6% of all CTC-positive patients) with pleural metastases. In the CTC-negative group, four breast cancer-related deaths and three deaths by other causes occurred (6% and 5%, respectively, of all CTC-negative patients). The patients with breast cancer-related death had pleural, skin, bone and liver metastases. There was no significant difference in overall- and breast cancer-related survival between the CTC-positive and CTC-negative group (Fisher's exact test and Pearson's *χ*^2^-test: *P*=1.000 and *P*=0.686, respectively; Table 3). CTC positivity was not associated with breast cancer-related or overall survival with a hazard ratio of 1.02 (95% CI: 0.11–9.09, *P*=0.989) and 0.57 (95% CI: 0.07–4.63, *P*=0.594), respectively. The 4-year overall survival rates were 91 and 94% for CTC-negative and CTC-positive patients, respectively.

### Additional analyses: effect of frozen storage and the value of repeated sampling after therapy

We assessed the effect of frozen storage in eight early-stage and one advanced breast cancer patients and five healthy controls. All samples had a concordant CTC status in both the fresh and stored sample.

We also used the assay in peripheral blood samples of a subset of patients who were CTC negative at diagnosis and had remained disease free. Of the 45 women who participated, 40 women tested CTC negative and 5 women (5 out of 45 (11%)) tested CTC positive, currently without experiencing a diagnosed relapse of disease. During follow-up (median time from second blood collection to last follow-up: 6.2 months; range: 0.0–13) no relapses or deaths occurred.

## Discussion

An optimised CTC assay was used to estimate circulating tumour cell load in prospectively collected peripheral blood samples of 82 early-stage and 16 advanced breast cancer patients and 45 healthy female controls. In early-stage breast cancer patients, the sensitivity of our assay was 20%. Based on the data from several published studies with early-stage breast cancer patients (Pierga *et al*, 2008; Rack *et al*, 2008; Daskalaki *et al*, 2009; Xenidis *et al*, 2009; Krishnamurthy *et al*, 2010; Riethdorf *et al*, 2010) the assay's sensitivity was found to be comparable with other assays with high specificity, except for Daskalaki *et al* (2009) who found a higher sensitivity of 52.4% with a specificity of 97.8% in a study that had only included stage I and II breast cancer patients. The CellSearch system (Veridex, Warren, NJ, USA) is one of the most used and validated commercial CTC detection platforms currently available (Allard *et al*, 2004; Balic *et al*, 2005; Cristofanilli *et al*, 2007; Riethdorf *et al*, 2007; Krishnamurthy *et al*, 2010). This method was used in the recent GeparQuattro clinical trial where CTCs were prospectively monitored in neo-adjuvant therapy. Here, at baseline a sensitivity of 21.6% was found for CTC positivity, using a cutoff of ⩾1 CTC/7.5 ml peripheral blood (Riethdorf *et al*, 2010). While generally demonstrating a similar sensitivity and specificity to the CellSearch system, our assay offers several other advantages: first, our assay is objective, as an algorithm generates a single score automatically for determining CTC positivity based on tumour marker gene expression levels detected by QPCR. This is in contrast to the CellSearch system, in which images of cells are determined to be CTCs by software and need to be manually confirmed by an operator. This step can introduce subjectivity in scoring, which is significant when as few as one or two cells in a sample are sufficient to class the patient as ‘CTC-positive’. Recently, Kraan *et al* (2011) observed that image interpretation was the main contributor to between-laboratory variation. In addition, our assay is considerably less expensive, costing less than US$25 per sample, compared with approximately US$600 per sample for the CellSearch system (Kaiser, 2010). Finally, there already exists an equipment for the automation of the enrichment, cDNA preparation and QPCR steps in our assay, and hence it could potentially require little manual work or technical knowledge. Although this is a preliminary study and further validation involving additional patients would be required to confirm that an automated system could provide equivalent prognostic data, there is a potential for the use of such system in a clinical setting.

The threshold for QDA positivity was set at the highest QDA value in the healthy controls group, resulting in 100% specificity, as previously described (Bosma *et al*, 2002; Weigelt *et al*, 2003). Although our assay's sensitivity could have been augmented by lowering the threshold, priority was given to avoid false positives.

In total, 81% of the advanced patients assayed were CTC positive *vs* 0% of the healthy controls (Figure 1). We hypothesised that enriching a sample for tumour cells before assessing tumour marker gene expression would be beneficial for assay sensitivity, and this appears to be the case. We had previously demonstrated that when tumour cell enrichment was not performed, 30% of advanced patients assayed from a similarly selected patient group had a positive QDA score indicating CTC positivity *vs* 0% of healthy controls (Weigelt *et al*, 2003). We also had previously shown that using a positive enrichment strategy for cells expressing both EpCam and ErbB2 antigens resulted in the detection of higher levels of tumour marker gene expression than enriching for cells expressing just one of the antigens (Molloy *et al*, 2008). Also, the multimarker gene expression panel used has obvious benefits over the use of a single marker for the detection of tumour cells. Finally, the use of the quadratic discriminant score function is not prone to subjectivity in scoring or inaccuracies in quantitation as immunohistochemical staining or densitometry of an electrophoresed nucleic acid band can be.

Our platform also has limitations using immunomagnetic bead selection with antibodies directed at EpCam and ErbB2. This may very well also have caused a bias by not selecting any potential EpCam and ErbB2 non- or low-expressing cells. It is believed that CTCs may lose EpCam in order to intravasate and to reach circulation, in a process called epithelial-to-mesenchymal transition (EMT) (Bonnomet *et al*, 2010). Indeed, Sieuwerts *et al* (2009) showed that a subtype of breast cancer cells is not detected in the EpCam-based CellSearch assay. Consistent with this hypothesis, in the present study, blood samples of 17 early-stage *EGP*-negative patients were found positive for one or more of the other three marker genes (data not shown). These studies and our data confirm that indeed there may be phenotypic differences between CTCs.

Our previous study using non-tumour cell-enriched blood samples from advanced patients demonstrated that CTC status was an independent predictor of both progression-free and overall survival (Weigelt *et al*, 2003). The CTC positivity as determined by our assay was a significant predictor of relapse-free survival in the primary breast cancer patient cohort (multivariate HR=5.13, *P*=0.006, 95% CI: 1.62–16.31; Table 5). Importantly, multivariate analyses demonstrated that CTC status provided significant prognostic information that was independent of other commonly used clinical variables, including age at diagnosis, lymph node status, histological grade, hormone receptor status and HER2 status (Tables 4 and 5). Tumour size, which is known for its prognostic value, did not reach statistical significance in our analysis. In contrast to predicting for relapse, CTC positivity was not a predictor for breast cancer-related or overall survival. Others have demonstrated a significantly reduced survival for CTC-positive early-stage breast cancer patients (Rack *et al*, 2008; Daskalaki *et al*, 2009; Xenidis *et al*, 2009). The observation that CTC positivity did not predict for survival in the present study may be because of the combination of a follow-up time that was relatively short for the observation of deaths and the occurrence of a limited number of events, being eight deaths in total.

Additional exploratory analyses of our assay on the effect of frozen storage, which found concordant results in all samples, indicated the possibility to store freshly collected samples for a longer period and to process them later. If confirmed on a larger scale, this method would greatly facilitate sample collection and would present another advantage to other methods described earlier (Daskalaki *et al*, 2009), including the CellSearch system in which samples are required to be processed within 72 h (Riethdorf *et al*, 2007). The analysis on the value of repeated sampling after therapy did not show relevance during the relatively short follow-up, and the prospective value of this second sampling will be studied in the future in ongoing follow-up.

In conclusion, we have demonstrated a sensitive and specific platform for the detection of circulating tumour cells in both advanced and early-stage breast cancer patients. In this study with early-stage breast cancer patients, we found that our assay was prognostic for relapse-free survival. Further work will be required in prospective trials to fully determine whether our assay can be used to improve disease outcome in patients who are CTC positive.

## Figures and Tables

**Table 1:** Primer sequences

**Gene**	**GenBank accession**	**Sequence**	**Probe (5′FAM–3′TAMRA)**
*p1B*	L15203	Sense: CTGAGGAGTACGTGGGCCTG	CTGCAAACCAGTGTGCCGTGCC
		Antisense: AGTCCACCCTGTCCTTGGC	
*CK19*	M002276	Sense: CTACAGCCACTACTACACGAC	CACCATTGAGAACTCCAGGATTGTCCTGC
		Antisense: CAGAGCCTGTTCCGTCTCAAA	
*EGP*	M32306	Sense: CAGTTGGTGCACAAAATACTGTCA	TTGCTCAAAGCTGGCTGCCAAATGTT
		Antisense: CCATTCATTTCTGCCTTCATCA	
*MmGl*	AF015224	Sense: TTCTTAACCAAACGGATGAAACTCT	TGCTGTCATATATTAATTGCATAAACACCTCAACA
		Antisense: GGTCTTGCAGAAAGTTAAAATAAATCAC	TTG

Abbreviations: *p1B*=*human secretory protein p1.B*; *CK19*=*cytokeratin 19*; *EGP*=*human epithelial glycoprotein*; *MmGl*=*mammaglobin*.

**Table 2:** Clinical characteristics of stage I–III breast cancer patients (*n*=82), according to the CTC status

**Patients (*n*)**	**Total (%), *n*=82**	**CTC- negative (within group %), *n*=66**	**CTC- positive (within group %), *n*=16**	***P*-value**
*Age (years)*				
⩽45	12 (15)	9 (14)	3 (19)	0.694
>45	70 (85)	57 (86)	13 (81)	
				
*Stage of disease* a				
I	47 (57)	39 (59)	8 (50)	0.285
II	23 (28)	19 (29)	4 (25)	
III	12 (15)	8 (12)	4 (25)	
				
*Tumour size* a				
T1	62 (76)	51 (77)	11 (69)	0.200
T2	14 (17)	12 (18)	2 (13)	
T3	3 (4)	1 (2)	2 (13)	
T4	3 (4)	2 (3)	1 (6)	
				
*Node stage* a				
N0	60 (73)	48 (73)	12 (75)	0.785
N1	17 (21)	14 (21)	3 (19)	
N2	3 (4)	2 (3)	1 (6)	
N3	2 (2)	2 (3)	0 (0)	
				
*Histological grade*				
Grade 1	26 (32)	22 (33)	4 (25)	0.329
Grade 2	38 (46)	31 (47)	7 (44)	
Grade 3	18 (22)	13 (20)	5 (31)	
				
*Hormone receptor*				
Negative	9 (11)	7 (11)	2 (13)	1.00
ER and/or PR positive	72 (88)	58 (88)	14 (88)	
Unknown^b^	1 (1)	1 (2)	0 (0)	
				
*HER2 status* c				
Negative	63 (77)	52 (79)	11 (69)	0.332
Positive	18 (22)	13 (20)	5 (31)	
Unknown^b^	1 (1)	1 (2)	0 (0)	
				
*Adjuvant therapy*				
None	14 (17)	9 (14)	5 (31)	0.581
CT	1 (1)	1 (2)	0 (0)	
RT	25 (31)	22 (33)	3 (19)	
HT	3 (4)	2 (3)	1 (6)	
CT+RT	13 (16)	10 (15)	3 (19)	
RT+HT	11 (13)	10 (15)	1 (6)	
CT+RT+HT	15 (18)	12 (18)	3 (19)	
				
*Any adjuvant therapy*				
No	14 (17)	9 (14)	5 (31)	0.134
Yes	68 (83)	57 (86)	11 (69)	
				
*Trastuzumab therapy*				
No	68 (83)	54 (82	14 (88)	0.726
Yes	14 (17)	12 (18)	2 (12)	

Abbreviations: CTC-positive or CTC-negative=positive or negative circulating tumour cell status according to quadratic discriminant analysis (QDA) score; ER=oestrogen receptor; PR=progesterone receptor; HER2=human epidermal growth factor receptor 2; CT=chemotherapy; RT=radiotherapy; HT=hormonal therapy.

a TNM 6 classification according to the Union Internationale Contre le Cancer criteria.

b One patient with unknown hormone receptor and HER2 status was excluded from Fisher's exact test.

c HER2 positivity=3+ in immunohistochemistry or positive fluorescent *in situ* hybridisation test (FISH).

**Table 3:** Incidence of relapses and overall and breast cancer-related deaths in early breast cancer patients, in total and according to CTC status at diagnosis

**Patients (*n*)**	**Total (%), *n***=**82**	**CTC- negative (within group %), *n*=66**	**CTC- positive (within group %), *n*=16**	***P*-value**
*Relapse of disease*				
No	70 (85)	60 (91)	10 (63)	**0.010**
Yes	12 (15)	6 (9)	6 (38)	
				
*Overall survival*				
Alive	74 (90)	59 (89)	15 (94)	1.000
Death	8 (10)	7 (11)	1 (6)	
				
*Breast cancer-related survival*				
Alive	74 (90)	59 (89)	15 (94)	0.686
Death, breast cancer related	5 (6)	4 (6)	1 (6)	
Death, other cause	3 (4)	3 (5)	0 (0)	

Abbreviation: CTC-positive or CTC-negative=positive or negative circulating tumour cell status according to quadratic discriminant analysis (QDA) score.

Significant *P*-values are shown in bold.

**Table 4:** Univariate analysis of relapse-free survival by CTC positivity and common clinical variables

**Parameter**	**Hazard** **ratio**	**95**% **CI**	***P***-**value**	**Cox** **regression**: ***P***-**value**
CTC (positive *vs* negative), *n*=82	4.72	1.52–14.66	**0**.**007**	**0**.**003**
Age (<45 years *vs* >45 years), *n*=82	1.22	0.27–5.58	0.797	0.797
Tumour size (⩾T2 *vs* T1), *n*=82^a^	3.40	1.10–10.55	**0**.**034**	**0**.**025**
Node stage (N+ *vs* N0), *n*=82^a^	1.40	0.42–4.65	0.586	0.584
Histological grade (grade 3 *vs* 1 and 2), *n*=82	1.19	0.32–4.39	0.798	0.798
Hormone receptor negative *vs* positive, *n*=81	3.04	0.82–11.25	0.096	0.080
HER2 positive *vs* negative, *n*=81	1.17	0.32–4.31	0.818	0.817

Abbreviations: CI=confidence interval; CTC-positive=positive circulating tumour cell status according to quadratic discriminant analysis (QDA) score; HER2=human epidermal growth factor receptor 2.

a TNM 6 classification according to the Union Internationale Contre le Cancer criteria.

Significant *P*-values are shown in bold.

**Table 5:** Multivariate analysis for relapse-free survival

**Parameter**	**Hazard ratio**	**95% CI**	***P*-value**
CTC-positive	5.13	1.62–16.31	**0.006**
Tumour size (⩾T2)^a^	3.11	0.99–9.72	0.051
Hormone receptor (negative)	2.93	0.77–11.14	0.116

Abbreviations: CI=confidence interval; CTC-positive=positive circulating tumour cell status according to quadratic discriminant analysis (QDA) score.

a TNM 6 classification according to the Union Internationale Contre le Cancer criteria.

Significant *P*-values are shown in bold.
